# *In vitro* activity of the novel β-lactamase inhibitor nacubactam against a nationwide collection of carbapenem-resistant Enterobacterales in Japan

**DOI:** 10.1093/jacamr/dlag096

**Published:** 2026-06-03

**Authors:** Katsunori Yanagihara, Kazuhiro Tateda, Yohei Doi, Satoshi Takahashi, Hiroki Ohge, Koju Itahashi, Hayato Okade, Hiroshige Mikamo

**Affiliations:** Department of Laboratory Medicine, Nagasaki University Graduate School of Biomedical Sciences, Nagasaki, Japan; Department of Microbiology and Infectious Diseases, Toho University School of Medicine, Tokyo, Japan; Departments of Microbiology and Infectious Diseases, Fujita Health University School of Medicine, Aichi, Japan; Division of Infectious Diseases, University of Pittsburgh School of Medicine, Pittsburgh, PA, USA; Department of Infection Control and Laboratory Medicine, Sapporo Medical University School of Medicine, Sapporo, Japan; Department of Infectious Diseases, Hiroshima University Hospital, Hiroshima, Japan; R&D Division, Meiji Seika Pharma Co., Ltd, Tokyo, Japan; R&D Division, Meiji Seika Pharma Co., Ltd, Tokyo, Japan; Department of Clinical Infectious Diseases, Aichi Medical University, Aichi, Japan

## Abstract

**Objectives:**

Nacubactam is a novel diazabicyclooctane carbapenemase inhibitor that, in combination with cefepime or aztreonam, has been submitted for regulatory approval in Japan for treating serious Gram-negative infections. This study aimed to evaluate the *in vitro* activity of nacubactam combined with cefepime or aztreonam against a nationwide collection of clinical carbapenem-resistant Enterobacterales (CRE) isolates in Japan.

**Methods:**

Minimum inhibitory concentration (MIC) values of 376 CRE isolates from 6 medical institutions in Japan were determined according to the Clinical and Laboratory Standards Institute (CLSI) methods. Results were interpreted using clinical breakpoints of the CLSI document M100 (2025) and the European Committee on Antimicrobial Susceptibility Testing clinical breakpoints, version 15.0 (2025).

**Results:**

The predominant CRE isolates were *Klebsiella pneumoniae* (*n* = 98), *Klebsiella aerogenes* (*n* = 62) and *Escherichia coli* (*n* = 43), with the *Enterobacter cloacae* complex accounting for 117 isolates. Carbapenemase-producing Enterobacterales (CPE) accounted for 48.9% (*n* = 184), with the predominant carbapenemases being IMP-1 (*n* = 70), IMP-6 (*n* = 43) and NDM (*n* = 17). Cefepime–nacubactam and aztreonam–nacubactam exhibited potent antibacterial activity against CRE isolates with MIC_50/90_ of 1/4 and 0.5/2 mg/L, respectively. Cefepime–nacubactam and aztreonam–nacubactam demonstrated potent activity against CPE, with MIC_50/90_ of 2/4 and 0.5/2 mg/L, respectively, which were lower than those for ceftazidime–avibactam (64/>64 mg/L) and imipenem–relebactam (2/16 mg/L). Both combinations exhibited potent antibacterial activity against non-carbapenemase-producing carbapenem-resistant Enterobacterales (non-CP-CRE), with MIC_50/90_ of 0.25/4 and 0.5/4 mg/L, respectively.

**Conclusions:**

Cefepime–nacubactam and aztreonam–nacubactam have excellent antibacterial activities against CPE and non-CP-CRE, supporting their potential as therapeutic options for CRE infections.

## Introduction

The emergence of carbapenem-resistant bacteria remains a serious problem.^1^ Carbapenem resistance is often mediated by carbapenemase production. Two key strategies to counter β-lactamase-mediated resistance are (1) the development of β-lactam antibiotics that evade enzymatic degradation and (2) the design of β-lactamase inhibitors that protect β-lactams from inactivation.^2,3^ Examples of the former include cefiderocol, whereas the latter include novel β-lactamase inhibitors such as avibactam and relebactam. Currently, resistance has been reported in strains with specific mutations, including alterations in the CirA receptor and PBP3 that affect cefiderocol,^4,5^ in metallo-β-lactamase (MBL)-producing strains, which are intrinsically not susceptible to ceftazidime–avibactam and imipenem-cilastatin–relebactam,^6,7^ and in OmpK36-mutated strains that develop resistance to these agents.^6–8^ Additionally, aztreonam–avibactam, which was recently approved in Europe (2024)^9^ and the USA (2025),^10^ showed reduced *in vitro* activity against Enterobacterales harbouring penicillin-binding protein (PBP) 3 insertion mutations in conjunction with either CMY-type AmpC production^11^ or NDM MBL production.^12^ These emerging resistance mechanisms highlight the urgent need for novel agents with minimal cross-resistance.

Nacubactam (formerly OP0595/RG6080) is a novel diazabicyclooctane derivative that has been evaluated in combination with β-lactam antibiotics. Unlike conventional β-lactamase inhibitor such as avibactam and relebactam,^3^ nacubactam possesses a unique dual mechanism of action: (i) inhibition of serine β-lactamases, including Ambler Class A (e.g. ESBLs, KPC), Class C (AmpC), and some in Class D enzymes, thereby protecting the partner β-lactam from enzymatic degradation and (ii) direct antibacterial activity through high-affinity binding to PBP2 of Enterobacterales, thereby enhancing the antimicrobial activity of partner β-lactam agents that preferentially target PBP3.^13–15^ Importantly, nacubactam does not inhibit Class B MBLs, but its PBP2-binding activity distinguishes it from other diazabicyclooctane inhibitors such as avibactam and relebactam.^3,15^ Recently, Meiji Seika Pharma Co., Ltd. has completed 2 pivotal phase 3 trials, Integral-1 and Integral-2. Integral-1 assessed the efficacy and safety of cefepime–nacubactam or aztreonam–nacubactam compared with that of imipenem–cilastatin in patients with complicated urinary tract infections or acute uncomplicated pyelonephritis (ClinicalTrials.gov ID NCT05887908). Integral-2 assessed the efficacy and safety of cefepime–nacubactam and aztreonam–nacubactam compared with that of the best available therapy for adults with infections caused by carbapenem-resistant Enterobacterales (CRE) (ClinicalTrials.gov ID NCT05905055). Based on the results of these trials, an application for marketing approval has been submitted in Japan.^16^

In this study, we evaluated the *in vitro* antibacterial activity of cefepime–nacubactam (FNC) and aztreonam–nacubactam (ANC) against clinical CRE isolates collected from 6 medical institutions in Japan.

## Materials and methods

### Isolates

A total of 376 CRE isolates were collected from 6 medical institutions in Japan: Nagasaki University, Aichi Medical University, Toho University, Fujita Health University, Sapporo Medical University, and Hiroshima University, based on a minimum inhibitory concentration (MIC) of meropenem or imipenem of ≥2 mg/L (Table 1). The collected strains included *Escherichia coli* (*n* = 43), *Klebsiella pneumoniae* (*n* = 98), *Klebsiella aerogenes* (*n* = 62) and other *Klebsiella* spp. (*n* = 23), *Enterobacter cloacae* complex (*n* = 117), *Enterobacter* spp. (*n* = 1), *Citrobacter* spp. (*n* = 10), *Morganella morganii* (*n* = 2), *Proteus mirabilis* (*n* = 8), *Proteus vulgaris* (*n* = 1), *Proteus vulgaris*/*penneri* (*n* = 1), *Providencia stuartii* (*n* = 1) and *Serratia marcescens* (*n* = 9). Carbapenemase analysis was conducted at each institution by immunochromatography, multiplex polymerase chain reaction, or whole-genome sequencing. Among the collected strains, 156 produced MBLs (Class B), 28 produced serine-type carbapenemases (Class A or Class D), and 192 were non-carbapenemase producers.

**Table 1.** Mechanism-stratified distribution of 376 carbapenem-resistant Enterobacterales isolates from Japan by carbapenemase status/type and species

Enterobacterales (isolates)		Carbapenem-resistant Enterobacterales^a^ (376 isolates)						
		CPE/CRE (184)						Non-CPE/CRE (192)
		MBL-producing Enterobacterales (156)					Serine β-lactamase^b^ (28)	
		IMP-1 (70)	IMP-6 (43)	Non- identified IMP (22)	NDM (17)	Unknown MBL (4)		
Enterobacteriaceae (354)	*Escherichia coli* (43)	3	14	2	5	—	3	16
	*Klebsiella pneumoniae* (98)	27	17	5	9	—	12	28
	*Klebsiella aerogenes* (62)	—	—	—	—	—	2	60
	Other *Klebsiella* spp. (23)	11	5	—	—	—	1	6
	*Enterobacter cloacae* complex (117)	24	5	14	2	2	9	61
	*Enterobacter* spp. (1)	—	—	—	—	—	—	1
	*Citrobacter* spp. (10)	4	2	1	—	—	—	3
Morganellaceae (13)	*Morganella morganii* (2)	—	—	—	—	—	—	2
	*Proteus mirabilis* (8)	—	—	—	—	—	—	8
	*Proteus vulgaris* (1)	—	—	—	—	1	—	—
	*Proteus vulgaris*/*penneri* (1)	—	—	—	—	—	—	1
	*Providencia stuartii* (1)	—	—	—	—	—	—	1
Yersiniaceae (9)	*Serratia marcescens* (9)	1	—	—	1	1	1	5

CPE, carbapenemase-producing Enterobacterales; CRE, carbapenem-resistant Enterobacterales; MBL, metallo-β-lactamase.

^a^CREs were defined as isolates with imipenem and/or meropenem MIC ≥2 mg/L.

^b^OXA-48 (8), KPC (14), NMC or NNMC/IMI (4), GES (1) and *mCIM*-positive cells (1).

### MIC determination

The antibacterial activity of FNC and ANC (both tested at a 1:1 concentration ratio; β-lactam concentration range, 0.03 to 64 mg/L) was measured using frozen 96-well plates from Eiken Chemical Co., Ltd. (Tokyo, Japan) and evaluated by broth microdilution according to the 2018 Clinical and Laboratory Standards Institute (CLSI) guidelines. As comparators, ceftazidime–avibactam (0.03–64 mg/L), aztreonam–avibactam (0.03–64 mg/L), imipenem–relebactam (0.03–64 mg/L), cefepime (0.06–128 mg/L), aztreonam (0.06–128 mg/L), piperacillin–tazobactam (0.06–128 mg/L), ceftazidime (0.03–64 mg/L), cefotaxime (0.06–128 mg/L), imipenem (0.06–64 mg/L), meropenem (0.06–64 mg/L), amikacin (0.06–128 mg/L), levofloxacin (0.06–128 mg/L) and colistin (0.25–4 mg/L) were used. The antibacterial activity of fosfomycin (0.12–128 mg/L), for which the CLSI recommends the agar dilution method, was, however, measured by broth microdilution for reference. For ceftazidime–avibactam, aztreonam–avibactam, imipenem–relebactam and piperacillin–tazobactam, inhibitor concentrations were fixed at 4 mg/L. For all β-lactam–inhibitor combinations including FNC and ANC, MICs were expressed as the β-lactam concentration. Additionally, 25 mg/L glucose-6-phosphate was added to the fosfomycin medium. Nacubactam alone was also tested separately, and MICs were reported as the nacubactam concentration. Measurements were conducted at each participating institution, with *E. coli* ATCC 25922, *K. pneumoniae* ATCC 700603 and *P. aeruginosa* ATCC 27853 used as quality control strains. All quality control results were within the acceptable ranges specified in CLSI M100 (2025), confirming the validity of the testing procedure. Carbapenem-resistant quality control strains were not used, as standard susceptible reference strains adequately validate the technical performance of the broth microdilution method for all agents tested. Susceptibility breakpoints were utilized according to the 2025 CLSI guidelines and the European Committee on Antimicrobial Susceptibility Testing (EUCAST) Clinical Breakpoints, version 15.0 (2025), to determine susceptibility rates. For agent–organism combinations without established breakpoints (including cefepime–nacubactam, aztreonam–nacubactam, aztreonam–avibactam, nacubactam and colistin versus certain Enterobacterales), results were not interpreted.

## Results

The most common species were *K. pneumoniae* (*n* = 98), *K. aerogenes* (*n* = 62) and *E. coli* (*n* = 43); additionally, the *E. cloacae* complex comprised 117 isolates. In total, 376 CRE isolates comprised 184 carbapenemase-producing Enterobacterales (CPE) and 192 non-carbapenemase-producing CRE (non-CP-CRE), with 84.8% (*n* = 156) of the CPE being MBL producers. The major carbapenemase types were IMP-1 (*n* = 70, 42.6%), followed by IMP-6 (*n* = 43, 23.3%), unidentified IMP (*n* = 22, 12.0%) and NDM (*n* = 17, 9.2%; Table 1).

FNC and ANC demonstrated potent antibacterial activity against 354 strains of CRE, with MIC_50/90_ values of 1/4 and 0.5/2 mg/L, respectively (Tables 2–4). Aztreonam–avibactam also showed strong activity (MIC_50/90_, 0.12/1 mg/L), whereas imipenem–relebactam and ceftazidime–avibactam showed moderate and limited activity, respectively (MIC_50/90_, 0.5/4 mg/L and 2/>64 mg/L, respectively).

**Table 2.** MIC distributions of cefepime–nacubactam and aztreonam–nacubactam against clinical carbapenem-resistant Enterobacterales (defined as an MIC ≥2 mg/L for either imipenem or meropenem) isolates from Japan

Organism	Antimicrobial agent	No. of isolates by MIC (mg/L) (cumulative % ≤ MIC)												
(No. of isolates)		≤0.03	0.06	0.12	0.25	0.5	1	2	4	8	16	32	64	>64
Enterobacterales	Cefepime–nacubactam	55	30	7	18	26	79	120	28	3	5	5	0	0
(376)		(14.6)	(22.6)	(24.5)	(29.3)	(36.2)	(57.2)	(89.1)	(96.5)	(97.3)	(98.7)	(100)	(100)	(100)
	Aztreonam–nacubactam	32	37	37	51	55	67	61	32	2	0	0	0	2
		(8.5)	(18.4)	(28.2)	(41.8)	(56.4)	(74.2)	(90.4)	(98.9)	(99.5)	(99.5)	(99.5)	(99.5)	(100)
Enterobacteriaceae	Cefepime–nacubactam	51	26	6	15	25	77	115	28	3	4	4	0	0
(354)		(14.4)	(21.8)	(23.4)	(27.7)	(34.7)	(56.5)	(89.0)	(96.9)	(97.7)	(98.9)	(100)	(100)	(100)
	Aztreonam–nacubactam	23	35	37	47	55	66	59	28	2	0	0	0	2
		(6.5)	(16.4)	(26.8)	(40.1)	(55.6)	(74.3)	(91.0)	(98.9)	(99.4)	(99.4)	(99.4)	(99.4)	(100)
*Escherichia coli*	Cefepime–nacubactam	0	0	0	1	5	19	12	6	0	0	0	0	0
(43)		(0)	(0)	(0)	(2.3)	(14.0)	(58.1)	(86.0)	(100)	(100)	(100)	(100)	(100)	(100)
	Aztreonam–nacubactam	0	1	2	8	11	6	8	7	0	0	0	0	0
		(0)	(2.3)	(7.0)	(25.6)	(51.2)	(65.1)	(83.7)	(100)	(100)	(100)	(100)	(100)	(100)
*Klebsiella pneumoniae*	Cefepime–nacubactam	2	0	0	2	11	32	31	13	3	3	1	0	0
(98)		(2.0)	(2.0)	(2.0)	(4.1)	(15.3)	(48.0)	(79.6)	(92.9)	(95.9)	(99.0)	(100)	(100)	(100)
	Aztreonam–nacubactam	1	4	5	14	16	31	19	7	1	0	0	0	0
		(1.0)	(5.1)	(10.2)	(24.5)	(40.8)	(72.4)	(91.8)	(99.0)	(100)	(100)	(100)	(100)	(100)
*Klebsiella aerogenes*	Cefepime–nacubactam	25	15	1	3	4	6	7	1	0	0	0	0	0
(62)		(40.3)	(64.5)	(66.1)	(71.0)	(77.4)	(87.1)	(98.4)	(100)	(100)	(100)	(100)	(100)	(100)
	Aztreonam–nacubactam	7	20	4	7	3	5	11	4	1	0	0	0	0
		(11.3)	(43.5)	(50.0)	(61.3)	(66.1)	(74.2)	(91.9)	(98.4)	(100)	(100)	(100)	(100)	(100)
Other *Klebsiella* spp.	Cefepime–nacubactam	0	0	0	1	0	5	15	2	0	0	0	0	0
(23)		(0)	(0)	(0)	(4.3)	(4.3)	(26.1)	(91.3)	(100)	(100)	(100)	(100)	(100)	(100)
	Aztreonam–nacubactam	0	0	3	3	9	3	3	2	0	0	0	0	0
		(0)	(0)	(13.0)	(26.1)	(65.2)	(78.3)	(91.3)	(100)	(100)	(100)	(100)	(100)	(100)
*Enterobacter cloacae* complex	Cefepime–nacubactam	24	10	5	7	5	14	43	5	0	1	3	0	0
(117)		(20.5)	(29.1)	(33.3)	(39.3)	(43.6)	(55.6)	(92.3)	(96.6)	(96.6)	(97.4)	(100)	(100)	(100)
	Aztreonam–nacubactam	14	10	22	14	13	19	15	8	0	0	0	0	2
		(12.0)	(20.5)	(39.3)	(51.3)	(62.4)	(78.6)	(91.5)	(98.3)	(98.3)	(98.3)	(98.3)	(98.3)	(100)
Morganellaceae	Cefepime–nacubactam	4	4	1	3	0	0	1	0	0	0	0	0	0
(13)		(30.8)	(61.5)	(69.2)	(92.3)	(92.3)	(92.3)	(100)	(100)	(100)	(100)	(100)	(100)	(100)
	Aztreonam–nacubactam	9	2	0	1	0	0	0	1	0	0	0	0	0
		(69.2)	(84.6)	(84.6)	(92.3)	(92.3)	(92.3)	(92.3)	(100)	(100)	(100)	(100)	(100)	(100)
Yersiniaceae (*Serratia marcescens*)	Cefepime–nacubactam	0	0	0	0	1	2	4	0	0	1	1	0	0
(9)		(0)	(0)	(0)	(0)	(11.1)	(33.3)	(77.8)	(77.8)	(77.8)	(88.9)	(100)	(100)	(100)
	Aztreonam–nacubactam	0	0	0	3	0	1	2	3	0	0	0	0	0
		(0)	(0)	(0)	(33.3)	(33.3)	(44.4)	(66.7)	(100)	(100)	(100)	(100)	(100)	(100)

MIC, minimum inhibitory concentration.

MICs for cefepime–nacubactam and aztreonam–nacubactam are expressed as the β-lactam concentration; both combinations were tested at a 1:1 concentration ratio.

**Table 3.** Mechanism-stratified MIC distributions of cefepime–nacubactam and aztreonam–nacubactam among clinical carbapenem-resistant Enterobacterales (defined as an MIC ≥2 mg/L for either imipenem or meropenem) isolates from Japan

Mechanism	Antimicrobial agent	No. of isolates by MIC (mg/L) (cumulative % ≤ MIC)												
(No. of isolates)		≤0.03	0.06	0.12	0.25	0.5	1	2	4	8	16	32	64	>64
Carbapenemase positive	Cefepime–nacubactam	2	2	0	7	17	57	78	10	3	5	3	0	0
(184)		(1.1)	(2.2)	(2.2)	(6.0)	(15.2)	(46.2)	(88.6)	(94.0)	(95.7)	(98.4)	(100)	(100)	(100)
	Aztreonam–nacubactam	3	9	20	35	49	47	20	1	0	0	0	0	0
		(1.6)	(6.5)	(17.4)	(36.4)	(63.0)	(88.6)	(99.5)	(100)	(100)	(100)	(100)	(100)	(100)
Metallo-β-lactamase	Cefepime–nacubactam	0	1	0	1	8	51	74	10	3	5	3	0	0
(156)		(0)	(0.6)	(0.6)	(1.3)	(6.4)	(39.1)	(86.5)	(92.9)	(94.9)	(98.1)	(100)	(100)	(100)
	Aztreonam–nacubactam	3	9	19	35	47	30	13	0	0	0	0	0	0
		(1.9)	(7.7)	(19.9)	(42.3)	(72.4)	(91.7)	(100)	(100)	(100)	(100)	(100)	(100)	(100)
IMP-1	Cefepime–nacubactam	0	0	0	0	2	18	44	5	1	0	0	0	0
(70)		(0)	(0)	(0)	(0)	(2.9)	(28.6)	(91.4)	(98.6)	(100)	(100)	(100)	(100)	(100)
	Aztreonam–nacubactam	0	2	11	16	17	17	7	0	0	0	0	0	0
		(0)	(2.9)	(18.6)	(41.4)	(65.7)	(90.0)	(100)	(100)	(100)	(100)	(100)	(100)	(100)
IMP-6	Cefepime–nacubactam	0	0	0	1	5	26	10	0	0	1	0	0	0
(43)		(0)	(0)	(0)	(2.3)	(14.0)	(74.4)	(97.7)	(97.7)	(97.7)	(100)	(100)	(100)	(100)
	Aztreonam–nacubactam	1	0	2	14	24	2	0	0	0	0	0	0	0
		(2.3)	(2.3)	(7.0)	(39.5)	(95.3)	(100)	(100)	(100)	(100)	(100)	(100)	(100)	(100)
Unidentified IMP	Cefepime–nacubactam	0	0	0	0	1	4	13	1	0	1	2	0	0
(22)		(0)	(0)	(0)	(0)	(4.5)	(22.7)	(81.8)	(86.4)	(86.4)	(90.9)	(100)	(100)	(100)
	Aztreonam–nacubactam	1	6	5	4	3	2	1	0	0	0	0	0	0
		(4.5)	(31.8)	(54.5)	(72.7)	(86.4)	(95.5)	(100)	(100)	(100)	(100)	(100)	(100)	(100)
NDM	Cefepime–nacubactam	0	0	0	0	0	3	5	4	2	3	0	0	0
(17)		(0)	(0)	(0)	(0)	(0)	(17.6)	(47.1)	(70.6)	(82.4)	(100)	(100)	(100)	(100)
	Aztreonam–nacubactam	0	0	1	1	2	9	4	0	0	0	0	0	0
		(0)	(0)	(5.9)	(11.8)	(23.5)	(76.5)	(100)	(100)	(100)	(100)	(100)	(100)	(100)
Unknown metallo-β-lactamase	Cefepime–nacubactam	0	1	0	0	0	0	2	0	0	0	1	0	0
(4)		(0)	(25.0)	(25.0)	(25.0)	(25.0)	(25.0)	(75.0)	(75.0)	(75.0)	(75.0)	(100)	(100)	(100)
	Aztreonam–nacubactam	1	1	0	0	1	0	1	0	0	0	0	0	0
		(25.0)	(50.0)	(50.0)	(50.0)	(75.0)	(75.0)	(100)	(100)	(100)	(100)	(100)	(100)	(100)
Non-metallo-β-lactamase	Cefepime–nacubactam	2	1	0	6	9	6	4	0	0	0	0	0	0
(28)		(7.1)	(10.7)	(10.7)	(32.1)	(64.3)	(85.7)	(100)	(100)	(100)	(100)	(100)	(100)	(100)
	Aztreonam–nacubactam	0	0	1	0	2	17	7	1	0	0	0	0	0
		(0)	(0)	(3.6)	(3.6)	(10.7)	(71.4)	(96.4)	(100)	(100)	(100)	(100)	(100)	(100)
KPC	Cefepime–nacubactam	0	0	0	3	9	1	1	0	0	0	0	0	0
(14)		(0)	(0)	(0)	(21.4)	(85.7)	(92.9)	(100)	(100)	(100)	(100)	(100)	(100)	(100)
	Aztreonam–nacubactam	0	0	0	0	1	8	5	0	0	0	0	0	0
		(0)	(0)	(0)	(0)	(7.1)	(64.3)	(100)	(100)	(100)	(100)	(100)	(100)	(100)
OXA-48	Cefepime–nacubactam	0	0	0	1	0	5	2	0	0	0	0	0	0
(8)		(0)	(0)	(0)	(12.5)	(12.5)	(75.0)	(100)	(100)	(100)	(100)	(100)	(100)	(100)
	Aztreonam–nacubactam	0	0	1	0	0	7	0	0	0	0	0	0	0
		(0)	(0)	(12.5)	(12.5)	(12.5)	(100)	(100)	(100)	(100)	(100)	(100)	(100)	(100)
Carbapenemase negative	Cefepime–nacubactam	53	28	7	11	9	22	42	18	0	0	2	0	0
(192)		(27.6)	(42.2)	(45.8)	(51.6)	(56.3)	(67.7)	(89.6)	(99.0)	(99.0)	(99.0)	(100)	(100)	(100)
	Aztreonam–nacubactam	29	28	17	16	6	20	41	31	2	0	0	0	2
		(15.1)	(29.7)	(38.5)	(46.9)	(50.0)	(60.4)	(81.8)	(97.9)	(99.0)	(99.0)	(99.0)	(99.0)	(100)

MIC, minimum inhibitory concentration.

MICs for cefepime–nacubactam and aztreonam–nacubactam are expressed as the β-lactam concentration; both combinations were tested at a 1:1 concentration ratio.

**Table 4.** MIC metrics (MIC_50_/MIC_90_/ranges) and susceptibility rates (CLSI and EUCAST) for antimicrobial agents, including nacubactam combinations, against CRE (defined as an MIC ≥2 mg/L for either imipenem or meropenem) in Japan, overall and by organism group/species

Organism	Antimicrobial agent	No.	MIC (mg/L)			CLSI	EUCAST
			MIC_50_	MIC_90_	Range	%S^a^	%S^a^
Enterobacterales	Cefepime–nacubactam^b^	376	1	4	≤0.03–32	NA	NA
	Cefepime	375	16	>128	≤0.06 to >128	29.3%	27.7%
	Aztreonam–nacubactam^b^	376	0.5	2	≤0.03 to >64	NA	NA
	Aztreonam–avibactam^c^	376	0.12	1	≤0.03 to >64	NA	NA
	Aztreonam	376	16	>128	≤0.06 to >128	42.3%	37.8%
	Ceftazidime–avibactam^c^	376	2	>64	≤0.03 to >64	62.2%	62.2%
	Ceftazidime	376	>64	>64	0.06 to >64	26.9%	22.6%
	Cefotaxime	376	128	>128	≤0.06 to >128	22.9%	22.9%
	Imipenem–relebactam^c^	376	0.5	4	0.06 to >64	67.8%	NA
	Imipenem	376	2	16	≤0.06 to >64	20.5%	NA
	Meropenem	376	4	16	≤0.06 to >64	29.8%	42.6%
	Piperacillin–tazobactam^c^	376	16	>128	0.12 to >128	45.5%	45.5%
	Amikacin	376	2	8	0.25 to >128	86.7%	90.2%
	Levofloxacin	376	0.5	64	≤0.06 to >128	50.5%	50.5%
	Fosfomycin^d^	376	16	>128	≤0.12 to >128	NA	NA
	Colistin	374	1	>4	≤0.25 to >4	NA	NA
	Nacubactam	376	4	>16	1 to >16	NA	NA
Enterobacteriaceae	Cefepime–nacubactam^b^	354	1	4	≤0.03–32	NA	NA
	Cefepime	353	16	>128	≤0.06 to >128	27.5%	25.8%
	Aztreonam–nacubactam^b^	354	0.5	2	≤0.03 to >64	NA	NA
	Aztreonam–avibactam^c^	354	0.12	1	≤0.03 to >64	NA	NA
	Aztreonam	354	32	>128	≤0.06 to >128	40.1%	36.2%
	Ceftazidime–avibactam^c^	354	2	>64	0.06 to >64	60.7%	60.7%
	Ceftazidime	354	>64	>64	0.06 to >64	24.0%	20.3%
	Cefotaxime	354	128	>128	≤0.06 to >128	21.2%	21.2%
	Imipenem–relebactam^c^	354	0.5	4	0.06 to >64	71.2%	83.9%
	Imipenem	354	2	16	≤0.06 to >64	21.8%	61.6%
	Meropenem	354	4	16	≤0.06 to >64	26.8%	40.1%
	Piperacillin–tazobactam^c^	354	16	>128	0.5 to >128	44.1%	44.1%
	Amikacin	354	2	8	0.25 to >128	89.0%	91.8%
	Levofloxacin	354	0.5	64	≤0.06 to >128	50.3%	50.3%
	Fosfomycin^d^	354	16	>128	≤0.12 to >128	NA	NA
	Colistin	352	0.5	>4	≤0.25 to >4	NA	85.2%
	Nacubactam	354	4	>16	1 to >16	NA	NA
*Escherichia coli*	Cefepime–nacubactam^b^	43	1	4	0.25–4	NA	NA
	Cefepime	43	>128	>128	4 to >128	0.0%	0.0%
	Aztreonam–nacubactam^b^	43	0.5	4	0.06–4	NA	NA
	Aztreonam–avibactam^c^	43	0.12	1	≤0.03–4	NA	NA
	Aztreonam	43	>128	>128	≤0.06 to >128	11.6%	7.0%
	Ceftazidime–avibactam^c^	43	4	>64	0.12 to >64	62.8%	62.8%
	Ceftazidime	43	>64	>64	8 to >64	0.0%	0.0%
	Cefotaxime	43	>128	>128	32 to >128	0.0%	0.0%
	Imipenem–relebactam^c^	43	0.5	8	0.06–32	76.7%	83.7%
	Imipenem	43	2	16	0.12–32	48.8%	67.4%
	Meropenem	43	8	32	0.5–64	2.3%	16.3%
	Piperacillin–tazobactam^c^	43	>128	>128	1 to >128	39.5%	39.5%
	Amikacin	43	4	128	2 to >128	74.4%	81.4%
	Levofloxacin	43	16	64	≤0.06–64	27.9%	27.9%
	Fosfomycin^d^	43	2	16	0.25 to >128	NA	NA
	Colistin	43	0.5	1	≤0.25–2	NA	100%
	Nacubactam	43	2	16	1 to >16	NA	NA
*Klebsiella pneumoniae*	Cefepime–nacubactam^b^	98	2	4	≤0.03–32	NA	NA
	Cefepime	97	32	>128	0.12 to >128	8.2%	7.2%
	Aztreonam–nacubactam^b^	98	1	2	≤0.03–8	NA	NA
	Aztreonam–avibactam^c^	98	0.25	1	≤0.03–16	NA	NA
	Aztreonam	98	>128	>128	≤0.06 to >128	26.5%	24.5%
	Ceftazidime–avibactam^c^	98	16	>64	0.12 to >64	46.9%	46.9%
	Ceftazidime	98	>64	>64	0.25 to >64	6.1%	4.1%
	Cefotaxime	98	>128	>128	≤0.06 to >128	4.1%	4.1%
	Imipenem–relebactam^c^	98	0.5	16	0.06–64	62.2%	70.4%
	Imipenem	98	2	32	≤0.06–64	32.7%	56.1%
	Meropenem	98	8	64	≤0.06 to >64	7.1%	21.4%
	Piperacillin–tazobactam^c^	98	>128	>128	2 to >128	27.6%	27.6%
	Amikacin	98	1	>128	0.25 to >128	83.7%	84.7%
	Levofloxacin	98	8	128	≤0.06 to >128	25.5%	25.5%
	Fosfomycin^d^	98	64	>128	≤0.12–>128	NA	NA
	Colistin	98	1	2	≤0.25–>4	NA	93.9%
	Nacubactam	98	8	>16	1–>16	NA	NA
*Klebsiella aerogenes*	Cefepime–nacubactam^b^	62	0.06	2	≤0.03–4	NA	NA
	Cefepime	62	0.12	16	≤0.06–128	71.0%	69.4%
	Aztreonam–nacubactam^b^	62	0.12	2	≤0.03–8	NA	NA
	Aztreonam–avibactam^c^	62	0.12	2	≤0.03–32	NA	NA
	Aztreonam	62	0.25	128	≤0.06 to >128	62.9%	59.7%
	Ceftazidime–avibactam^c^	62	0.25	2	0.06–16	98.4%	98.4%
	Ceftazidime	62	0.5	>64	0.12 to >64	62.9%	56.5%
	Cefotaxime	62	0.12	>128	≤0.06 to >128	59.7%	59.7%
	Imipenem–relebactam^c^	62	0.25	1	0.06–2	95.2%	100%
	Imipenem	62	2	8	2–16	0.0%	69.4%
	Meropenem	62	≤0.06	8	≤0.06–8	69.4%	69.4%
	Piperacillin–tazobactam^c^	62	4	128	2 to >128	66.1%	66.1%
	Amikacin	62	2	4	0.5–8	96.8%	100%
	Levofloxacin	62	≤0.06	1	≤0.06–4	88.7%	88.7%
	Fosfomycin^d^	62	16	64	1 to >128	NA	NA
	Colistin	61	0.5	1	≤0.25 to >4	NA	98.4%
	Nacubactam	62	>16	>16	2 to >16	NA	NA
Other	Cefepime–nacubactam^b^	23	2	2	0.25–4	NA	NA
*Klebsiella* spp.	Cefepime	23	8	>128	4 to >128	0.0%	0.0%
	Aztreonam–nacubactam^b^	23	0.5	2	0.12–4	NA	NA
	Aztreonam–avibactam^c^	23	0.25	1	≤0.03–1	NA	NA
	Aztreonam	23	16	>128	0.12 to >128	39.1%	26.1%
	Ceftazidime–avibactam^c^	23	64	>64	0.5 to >64	30.4%	30.4%
	Ceftazidime	23	>64	>64	2 to >64	8.7%	0.0%
	Cefotaxime	23	64	>128	2 to >128	0.0%	0.0%
	Imipenem–relebactam^c^	23	2	4	0.12–4	47.8%	87.0%
	Imipenem	23	2	16	0.12–32	21.7%	60.9%
	Meropenem	23	8	16	1–64	4.3%	17.4%
	Piperacillin–tazobactam^c^	23	16	>128	2 to >128	39.1%	39.1%
	Amikacin	23	2	4	0.5 to >128	91.3%	95.7%
	Levofloxacin	23	4	16	≤0.06–64	30.4%	30.4%
	Fosfomycin^d^	23	64	>128	2 to >128	NA	NA
	Colistin	23	0.5	1	≤0.25–1	NA	100%
	Nacubactam	23	>16	>16	2 to >16	NA	NA
*Enterobacter cloacae* complex	Cefepime–nacubactam^b^	117	1	2	≤0.03–32	NA	NA
	Cefepime	117	8	128	≤0.06 to >128	35.9%	33.3%
	Aztreonam–nacubactam^b^	117	0.25	2	≤0.03 to >64	NA	NA
	Aztreonam–avibactam^c^	117	0.25	2	≤0.03 to >64	NA	NA
	Aztreonam	117	4	>128	≤0.06 to >128	50.4%	47.0%
	Ceftazidime–avibactam^c^	117	2	>64	0.06 to >64	59.0%	59.0%
	Ceftazidime	117	>64	>64	0.06 to >64	30.8%	27.4%
	Cefotaxime	117	128	>128	≤0.06 to >128	27.4%	27.4%
	Imipenem–relebactam^c^	117	1	4	0.12 to >64	67.5%	86.3%
	Imipenem	117	2	16	0.12 to >64	12.8%	60.7%
	Meropenem	117	2	16	≤0.06–64	35.0%	53.8%
	Piperacillin–tazobactam^c^	117	16	>128	0.5 to >128	48.7%	48.7%
	Amikacin	117	2	4	0.5–128	95.7%	97.4%
	Levofloxacin	117	0.25	8	≤0.06 to >128	61.5%	61.5%
	Fosfomycin^d^	117	32	>128	0.5 to >128	NA	NA
	Colistin	116	1	>4	≤0.25 to >4	NA	62.1%
	Nacubactam	117	4	>16	1 to >16	NA	NA
*Citrobacter* spp.	Cefepime–nacubactam^b^	10	2	2	0.25–4	NA	NA
	Cefepime	10	16	>128	0.5 to >128	20.0%	10.0%
	Aztreonam–nacubactam^b^	10	0.5	2	≤0.03–2	NA	NA
	Aztreonam–avibactam^c^	10	0.25	0.5	≤0.03–1	NA	NA
	Aztreonam	10	64	>128	≤0.06 to >128	30.0%	20.0%
	Ceftazidime–avibactam^c^	10	32	>64	1 to >64	40.0%	40.0%
	Ceftazidime	10	64	>64	2 to >64	10.0%	0.0%
	Cefotaxime	10	>128	>128	1 to >128	10.0%	10.0%
	Imipenem–relebactam^c^	10	0.5	4	0.25–4	80.0%	80.0%
	Imipenem	10	2	4	0.25–4	40.0%	60.0%
	Meropenem	10	4	8	0.5–16	10.0%	30.0%
	Piperacillin–tazobactam^c^	10	32	>128	1 to >128	40.0%	40.0%
	Amikacin	10	2	16	0.5–16	70.0%	80.0%
	Levofloxacin	10	0.25	16	≤0.06–16	60.0%	60.0%
	Fosfomycin^d^	10	1	2	0.5–2	NA	NA
	Colistin	10	0.5	1	≤0.25–1	NA	100%
	Nacubactam	10	4	>16	2 to >16	NA	NA
Morganellaceae	Cefepime–nacubactam^b^	13	0.06	0.25	≤0.03–2	NA	NA
	Cefepime	13	0.12	4	≤0.06–4	84.6%	84.6%
	Aztreonam–nacubactam^b^	13	≤0.03	0.25	≤0.03–4	NA	NA
	Aztreonam–avibactam^c^	13	≤0.03	0.06	≤0.03–4	NA	NA
	Aztreonam	13	≤0.06	0.5	≤0.06–4	100%	92.3%
	Ceftazidime–avibactam^c^	13	0.12	0.25	≤0.03–2	100%	100%
	Ceftazidime	13	0.12	0.5	0.06–2	100%	92.3%
	Cefotaxime	13	≤0.06	16	≤0.06 to >128	76.9%	76.9%
	Imipenem–relebactam^c^	13	4	4	1–8	7.7%	NA
	Imipenem	13	2	4	2–8	0.0%	NA
	Meropenem	13	0.12	0.5	≤0.06–0.5	100%	100%
	Piperacillin–tazobactam^c^	13	0.25	2	0.12–4	100%	100%
	Amikacin	13	4	16	4–32	53.8%	76.9%
	Levofloxacin	13	≤0.06	32	≤0.06–32	53.8%	53.8%
	Fosfomycin^d^	13	16	128	0.5 to >128	NA	NA
	Colistin	13	>4	>4	>4 to >4	NA	NA
	Nacubactam	13	>16	>16	>16 to >16	NA	NA
(*Yersiniaceae Serratia*	Cefepime–nacubactam^b^	9	—	—	0.5–32	—	—
*marcescens*)	Cefepime	9	—	—	1–128	—	—
	Aztreonam–nacubactam^b^	9	—	—	0.25–4	—	—
	Aztreonam–avibactam^c^	9	—	—	0.12–4	—	—
	Aztreonam	9	—	—	0.25–64	—	—
	Ceftazidime–avibactam^c^	9	—	—	0.5 to >64	—	—
	Ceftazidime	9	—	—	0.5 to >64	—	—
	Cefotaxime	9	—	—	1 to >128	—	—
	Imipenem–relebactam^c^	9	—	—	1–64	—	—
	Imipenem	9	—	—	2 to >64	—	—
	Meropenem	9	—	—	0.12 to >64	—	—
	Piperacillin–tazobactam^c^	9	—	—	1 to >128	—	—
	Amikacin	9	—	—	4–32	—	—
	Levofloxacin	9	—	—	0.12–8	—	—
	Fosfomycin^d^	9	—	—	8 to >128	—	—
	Colistin	9	—	—	1 to >4	—	—
	Nacubactam	9	—	—	>16 to >16	—	—

MIC, minimum inhibitory concentration; NA, Not applicable; breakpoints not established; —, Not calculated because of insufficient number of isolates (<10).

^a^Criteria as published by the CLSI M100 (35th ed., 2025) and EUCAST Breakpoint Tables v15.0 (2025).

^b^MICs for cefepime–nacubactam and aztreonam–nacubactam are expressed as the β-lactam concentration; both combinations were tested at a 1:1 concentration ratio.

^c^For ceftazidime–avibactam, aztreonam–avibactam, imipenem–relebactam, and piperacillin–tazobactam, inhibitor concentrations were fixed at 4 mg/L; MICs are expressed as the β-lactam concentration.

^d^Fosfomycin MICs from broth microdilution reported without interpretation; only agar dilution results are valid for susceptibility testing per CLSI/EUCAST.

FNC and ANC exhibited strong activity against *E. coli* (*n* = 43), with 100% inhibition at ≤4 mg/L. For *K. pneumoniae* (*n* = 98), FNC and ANC showed robust activity (MIC_50/90_ values of 2/4 and 1/2 mg/L, respectively), with 100% inhibition at ≤32 mg/L and ≤8 mg/L, respectively. Notably, 92.9% and 99.0% of isolates were inhibited at ≤4 mg/L for FNC and ANC, respectively. Similarly, FNC and ANC exhibited strong activity (MIC_50/90_ of 0.06/2 mg/L and 0.12/2 mg/L, respectively) against *K. aerogenes* (*n* = 62), with 100% inhibition at ≤4 mg/L and ≤8 mg/L, respectively. The activity against the *E. cloacae* complex (*n* = 117) was also excellent, with MIC_50/90_ values of 1/2 mg/L and 0.25/2 mg/L for FNC and ANC, respectively.

The antibacterial activities of FNC and ANC against carbapenemase-producing bacteria are shown in Tables 5 and 6, respectively. Of these, 156 were MBL-producing strains, including those that produced IMP and NDM. FNC demonstrated strong antibacterial activity against these MBL-producing strains, with MIC_50/90_ values of 2/4 mg/L, which were more than 16-fold lower than those with cefepime and more than 2-fold lower than those with nacubactam alone. Similarly, ANC had MIC_50/90_ values of 0.5/1 mg/L, which were more than 32-fold lower than those of aztreonam and more than 8-fold lower than those of nacubactam alone. As expected, all NDM-producing strains showed MIC values of ≥64 mg/L for ceftazidime–avibactam and ≥4 mg/L for imipenem–relebactam, as these agents lack activity against MBL producers. In contrast, FNC (with MIC_50/90_ values of 4/16 mg/L, exhibiting 47.1% and 82.4% inhibition at concentrations of ≤2 mg/L and ≤8 mg/L, respectively) and ANC (with MIC_50/90_ values of 1/2 mg/L, demonstrating 100% inhibition at ≤2 mg/L) showed excellent antibacterial activity.

**Table 5.** Mechanism-stratified MIC_50_/MIC_90_ values and MIC ranges for antimicrobial agents, including nacubactam combinations, against CRE (defined as an MIC ≥2 mg/L for either imipenem or meropenem) in Japan

Mechanism	Antimicrobial agent	No.	MIC (mg/L)		
			MIC_50_	MIC_90_	Range
Carbapenemase producing	Cefepime–nacubactam^a^	184	2	4	≤0.03–32
	Cefepime	183	32	>128	≤0.06 to >128
	Aztreonam–nacubactam^a^	184	0.5	2	≤0.03–4
	Aztreonam–avibactam^b^	184	0.12	1	≤0.03–4
	Aztreonam	184	32	>128	≤0.06 to >128
	Ceftazidime–avibactam^b^	184	64	>64	0.06 to >64
	Ceftazidime	184	>64	>64	0.06 to >64
	Cefotaxime	184	>128	>128	0.25 to >128
	Imipenem–relebactam^b^	184	2	16	0.06 to >64
	Imipenem	184	4	32	≤0.06 to >64
	Meropenem	184	8	32	0.12 to >64
	Piperacillin–tazobactam^b^	184	16	>128	0.25 to >128
	Amikacin	184	2	16	0.5 to >128
	Levofloxacin	184	2	64	≤0.06 to >128
	Fosfomycin^c^	184	16	>128	≤0.12 to >128
	Colistin	184	1	>4	≤0.25 to >4
	Nacubactam	184	4	>16	1 to >16
Metallo-β-lactamase	Cefepime–nacubactam^a^	156	2	4	0.06–32
	Cefepime	155	32	>128	0.5 to >128
	Aztreonam–nacubactam^a^	156	0.5	1	≤0.03–2
	Aztreonam–avibactam^b^	156	0.12	1	≤0.03–4
	Aztreonam	156	16	>128	≤0.06 to >128
	Ceftazidime–avibactam^b^	156	>64	>64	0.06 to >64
	Ceftazidime	156	>64	>64	0.06 to >64
	Cefotaxime	156	>128	>128	2 to >128
	Imipenem–relebactam^b^	156	2	16	0.06 to >64
	Imipenem	156	2	16	≤0.06 to >64
	Meropenem	156	4	32	0.12 to >64
	Piperacillin–tazobactam^b^	156	16	>128	0.25 to >128
	Amikacin	156	2	128	0.5 to >128
	Levofloxacin	156	2	64	≤0.06 to >128
	Fosfomycin^c^	156	16	128	0.25 to >128
	Colistin	156	1	4	≤0.25 to >4
	Nacubactam	156	4	>16	1 to >16
IMP-1	Cefepime–nacubactam^a^	70	2	2	0.5–8
	Cefepime	70	16	128	1 to >128
	Aztreonam–nacubactam^a^	70	0.5	1	0.06–2
	Aztreonam–avibactam^b^	70	0.25	1	≤0.03–1
	Aztreonam	70	4	>128	≤0.06 to >128
	Ceftazidime–avibactam^b^	70	>64	>64	8 to >64
	Ceftazidime	70	>64	>64	8 to >64
	Cefotaxime	70	128	>128	2 to >128
	Imipenem–relebactam^b^	70	2	4	0.25 to >64
	Imipenem	70	2	8	0.25 to >64
	Meropenem	70	4	16	0.5–32
	Piperacillin–tazobactam^b^	70	32	>128	2 to >128
	Amikacin	70	2	8	0.5 to >128
	Levofloxacin	70	2	64	≤0.06 to >128
	Fosfomycin^c^	70	16	>128	0.5 to >128
	Colistin	70	0.5	1	≤0.25 to >4
	Nacubactam	70	4	>16	2 to >16
IMP-6	Cefepime–nacubactam^a^	43	1	2	0.25–16
	Cefepime	43	16	>128	0.5 to >128
	Aztreonam–nacubactam^a^	43	0.5	0.5	≤0.03–1
	Aztreonam–avibactam^b^	43	0.06	0.12	≤0.03–0.25
	Aztreonam	43	32	>128	≤0.06 to >128
	Ceftazidime–avibactam^b^	43	16	64	1 to >64
	Ceftazidime	43	64	>64	8 to >64
	Cefotaxime	43	>128	>128	16 to >128
	Imipenem–relebactam^b^	43	0.25	1	0.06–4
	Imipenem	43	0.5	2	≤0.06–4
	Meropenem	43	8	32	2–64
	Piperacillin–tazobactam^b^	43	8	16	1 to >128
	Amikacin	43	2	16	0.5 to >128
	Levofloxacin	43	4	32	≤0.06–64
	Fosfomycin^c^	43	8	>128	0.25 to >128
	Colistin	43	0.5	1	≤0.25–4
	Nacubactam	43	2	>16	1 to >16
Unidentified IMP	Cefepime–nacubactam^a^	22	2	16	0.5–32
	Cefepime	21	32	128	4 to >128
	Aztreonam–nacubactam^a^	22	0.12	1	≤0.03–2
	Aztreonam–avibactam^b^	22	0.06	0.5	≤0.03–2
	Aztreonam	22	0.25	64	≤0.06 to >128
	Ceftazidime–avibactam^b^	22	>64	>64	8 to >64
	Ceftazidime	22	>64	>64	32 to >64
	Cefotaxime	22	128	>128	16 to >128
	Imipenem–relebactam^b^	22	2	16	1–32
	Imipenem	22	4	16	1–64
	Meropenem	22	4	32	1–32
	Piperacillin–tazobactam^b^	22	8	128	4 to >128
	Amikacin	22	2	4	1–16
	Levofloxacin	22	0.12	4	≤0.06–16
	Fosfomycin^c^	22	2	64	0.5–128
	Colistin	22	1	>4	0.5 to >4
	Nacubactam	22	4	>16	1 to >16
NDM	Cefepime–nacubactam^a^	17	4	16	1–16
	Cefepime	17	>128	>128	32 to >128
	Aztreonam–nacubactam^a^	17	1	2	0.12–2
	Aztreonam–avibactam^b^	17	0.25	1	≤0.03–4
	Aztreonam	17	>128	>128	0.12 to >128
	Ceftazidime–avibactam^b^	17	>64	>64	>64 to >64
	Ceftazidime	17	>64	>64	>64 to >64
	Cefotaxime	17	>128	>128	>128 to >128
	Imipenem–relebactam^b^	17	32	64	4–64
	Imipenem	17	32	64	4–64
	Meropenem	17	32	64	2 to >64
	Piperacillin–tazobactam^b^	17	>128	>128	>128 to >128
	Amikacin	17	16	>128	2 to >128
	Levofloxacin	17	32	128	≤0.06–128
	Fosfomycin^c^	17	8	128	0.5 to >128
	Colistin	17	1	4	≤0.25 to >4
	Nacubactam	17	4	>16	1 to >16
Unknown metallo-β-lactamase	Cefepime–nacubactam^a^	4	—	—	0.06–32
	Cefepime	4	—	—	0.5–64
	Aztreonam–nacubactam^a^	4	—	—	≤0.03–2
	Aztreonam–avibactam^b^	4	—	—	≤0.03–1
	Aztreonam	4	—	—	≤0.06–32
	Ceftazidime–avibactam^b^	4	—	—	0.06 to >64
	Ceftazidime	4	—	—	0.06 to >64
	Cefotaxime	4	—	—	16 to >128
	Imipenem–relebactam^b^	4	—	—	1–64
	Imipenem	4	—	—	1–64
	Meropenem	4	—	—	0.12–16
	Piperacillin–tazobactam^b^	4	—	—	0.25 to >128
	Amikacin	4	—	—	2–16
	Levofloxacin	4	—	—	≤0.06–8
	Fosfomycin^c^	4	—	—	32 to >128
	Colistin	4	—	—	0.5 to >4
	Nacubactam	4	—	—	2 to >16
Non-metallo-β-lactamase^d^	Cefepime–nacubactam^a^	28	0.5	2	≤0.03–2
	Cefepime	28	32	>128	≤0.06 to >128
	Aztreonam–nacubactam^a^	28	1	2	0.12–4
	Aztreonam–avibactam^b^	28	0.25	1	≤0.03–2
	Aztreonam	28	>128	>128	0.25 to >128
	Ceftazidime–avibactam^b^	28	1	2	0.12–4
	Ceftazidime	28	64	>64	0.5 to >64
	Cefotaxime	28	128	>128	0.25 to >128
	Imipenem–relebactam^b^	28	0.5	32	0.12–64
	Imipenem	28	8	>64	2 to >64
	Meropenem	28	8	64	0.5 to >64
	Piperacillin–tazobactam^b^	28	>128	>128	2 to >128
	Amikacin	28	1	16	0.5–64
	Levofloxacin	28	4	64	≤0.06 to >128
	Fosfomycin^c^	28	32	>128	≤0.12 to >128
	Colistin	28	1	>4	≤0.25 to >4
	Nacubactam	28	4	>16	1 to >16
KPC	Cefepime–nacubactam^a^	14	0.5	1	0.25–2
	Cefepime	14	32	>128	4 to >128
	Aztreonam–nacubactam^a^	14	1	2	0.5–2
	Aztreonam–avibactam^b^	14	0.12	0.5	≤0.03–1
	Aztreonam	14	>128	>128	64 to >128
	Ceftazidime–avibactam^b^	14	1	1	0.25–2
	Ceftazidime	14	>64	>64	8 to >64
	Cefotaxime	14	128	>128	32 to >128
	Imipenem–relebactam^b^	14	0.25	0.5	0.12–0.5
	Imipenem	14	8	16	2–32
	Meropenem	14	8	32	0.5–64
	Piperacillin–tazobactam^b^	14	>128	>128	128 to >128
	Amikacin	14	1	16	1–64
	Levofloxacin	14	1	32	≤0.06–32
	Fosfomycin^c^	14	32	128	2 to >128
	Colistin	14	≤0.25	>4	≤0.25 to >4
	Nacubactam	14	4	>16	2 to >16
OXA-48	Cefepime–nacubactam^a^	8	—	—	0.25–2
	Cefepime	8	—	—	1 to >128
	Aztreonam–nacubactam^a^	8	—	—	0.12–1
	Aztreonam–avibactam^b^	8	—	—	0.06–1
	Aztreonam	8	—	—	0.25 to >128
	Ceftazidime–avibactam^b^	8	—	—	0.12–2
	Ceftazidime	8	—	—	4 to >64
	Cefotaxime	8	—	—	2 to >128
	Imipenem–relebactam^b^	8	—	—	2–64
	Imipenem	8	—	—	2–64
	Meropenem	8	—	—	1–64
	Piperacillin–tazobactam^b^	8	—	—	>128 to >128
	Amikacin	8	—	—	0.5–16
	Levofloxacin	8	—	—	1 to >128
	Fosfomycin^c^	8	—	—	≤0.12 to >128
	Colistin	8	—	—	0.5 to >4
	Nacubactam	8	—	—	1 to >16
Non-carbapenemase producing	Cefepime–nacubactam^a^	192	0.25	4	≤0.03–32
	Cefepime	192	2	>128	≤0.06 to >128
	Aztreonam–nacubactam^a^	192	0.5	4	≤0.03 to >64
	Aztreonam–avibactam^b^	192	0.25	2	≤0.03 to >64
	Aztreonam	192	4	>128	≤0.06 to >128
	Ceftazidime–avibactam^b^	192	0.5	2	≤0.03 to >64
	Ceftazidime	192	4	>64	0.06 to >64
	Cefotaxime	192	8	>128	≤0.06 to >128
	Imipenem–relebactam^b^	192	0.5	2	0.06–8
	Imipenem	192	2	8	0.25–32
	Meropenem	192	0.5	8	≤0.06–32
	Piperacillin–tazobactam^b^	192	8	>128	0.12 to >128
	Amikacin	192	2	4	0.25–128
	Levofloxacin	192	0.12	32	≤0.06 to >128
	Fosfomycin^c^	192	32	>128	0.5 to >128
	Colistin	190	1	>4	≤0.25 to >4
	Nacubactam	192	>16	>16	1 to >16

MIC, minimum inhibitory concentration; —, Not calculated because of insufficient number of isolates (<10).

^a^MICs for cefepime–nacubactam and aztreonam–nacubactam are expressed as the β-lactam concentration; both combinations were tested at a 1:1 concentration ratio.

^b^For ceftazidime–avibactam, aztreonam–avibactam, imipenem–relebactam, and piperacillin–tazobactam, inhibitor concentrations were fixed at 4 mg/L; MICs are expressed as the β-lactam concentration.

^c^Fosfomycin MICs from broth microdilution reported without interpretation; only agar dilution results are valid for susceptibility testing per CLSI/EUCAST.

^d^Non-metallo-β-lactamase: OXA-48 (8), KPC (14), NMC or NMC/IMI (4), GES (1), and *mCIM*-positive (1).

**Table 6.** Profiles of strains with reduced susceptibility to cefepime–nacubactam (MIC >8 mg/L) and aztreonam–nacubactam (MIC >4 mg/L)

Organism	Carbapenemase	MIC (mg/L)																
		Cefepime–nacubactam	Cefepime	Aztreonam–nacubactam	Aztreonam–avibactam	Aztreonam	Ceftazidime–avibactam	Ceftazidime	Cefotaxime	Imipenem–relebactam	Imipenem	Meropenem	Piperacillin–tazobactam	Amikacin	Levofloxacin	Fosfomycin	Colistin	Nacubactam
*Klebsiella pneumoniae*	IMP-6	16	>128	0.25	0.12	>128	64	64	>128	4	4	64	4	2	16	>128	0.5	>16
*Klebsiella pneumoniae*	IMP	16	64	0.25	0.25	0.25	>64	>64	128	16	32	32	32	1	0.12	64	0.5	>16
*Klebsiella pneumoniae*	IMP	32	128	0.25	0.25	0.25	>64	>64	>128	32	64	32	32	2	0.12	128	1	>16
*Klebsiella pneumoniae*	NDM	16	>128	1	0.25	>128	>64	>64	>128	32	32	64	>128	2	32	8	1	>16
*Enterobacter cloacae* complex	IMP	32	64	1	1	64	>64	>64	>128	2	8	4	128	4	2	64	8	>16
*Enterobacter cloacae* complex	NDM	16	>128	1	0.25	>128	>64	>64	>128	64	64	64	>128	16	128	64	2	4
*Enterobacter cloacae* complex	Negative	32	64	>64	>64	>128	>64	>64	>128	1	1	4	128	128	1	32	8	>16
*Enterobacter cloacae* complex	Negative	32	32	>64	>64	>128	>64	>64	>128	1	1	4	8	128	1	>128	ND	>16
*Serratia marcescens*	NDM-1	16	128	0.25	0.12	64	>64	>64	>128	64	64	64	8	16	1	16	8	>16
*Serratia marcescens*	Metallo-β-lactamase	32	64	2	1	16	>64	>64	>128	64	64	16	>128	16	8	>128	8	>16

MIC, minimum inhibitory concentration.

MICs for β-lactam–β-lactamase inhibitor combinations are expressed as the β-lactam concentration; for ceftazidime–avibactam, aztreonam–avibactam and imipenem–relebactam, the inhibitor was fixed at 4 mg/L. Cefepime–nacubactam and aztreonam–nacubactam were tested at a 1:1 concentration ratio. Nacubactam was tested alone; MICs are reported as the nacubactam concentration. ND, not determined.

Against 28 strains producing serine-type carbapenemases, including OXA-48 (*n* = 8), KPC (*n* = 14), NMC or NMC/IMI (*n* = 4), GES (*n* = 1) and *mCIM*-positive strains (*n* = 1), FNC and ANC exhibited strong activity, inhibiting the growth of all strains at ≤2 mg/L and ≤4 mg/L, respectively. The MIC_50/90_ values for FNC were 0.5/2 mg/L, which were more than 64-fold lower than those for cefepime and more than 8-fold lower than those for nacubactam alone. Similarly, ANC has MIC_50/90_ values of 1/2 mg/L, which were more than 128-fold lower than those for aztreonam and more than 4-fold lower than those for nacubactam alone. The serine-type carbapenemase-producing strains also exhibited low MICs to ceftazidime–avibactam and aztreonam–avibactam (MIC_50/90_, 1/2 mg/L and 0.25/1 mg/L, respectively). In contrast, imipenem–relebactam showed reduced activity (MIC_50/90_, 0.5/32 mg/L), with 11 of 28 strains (39.3%) showing MIC values ≥4 mg/L. Notably, among the 8 OXA-48-producing strains, 6 (75.0%) exhibited imipenem–relebactam MIC values ≥4 mg/L.

Among 192 non-carbapenemase-producing strains, FNC and ANC demonstrated strong antibacterial activity. FNC had MIC_50/90_ values of 0.25/4 mg/L, which were more than 8-fold lower than those for cefepime and nacubactam alone. ANC had MIC_50/90_ values of 0.5/4 mg/L, which were more than 8-fold lower than those for aztreonam and more than 4-fold lower than those for nacubactam alone.

Reduced susceptibility (MIC >8 mg/L for FNC; MIC >4 mg/L for ANC) was observed in 10 strains for FNC and 2 strains for ANC (Table 6). Reduced susceptibility to FNC was observed in 4 MBL-producing *K. pneumoniae*, 2 MBL-producing *E. cloacae* complex, 2 MBL-producing *S. marcescens* and 2 carbapenemase-non-producing *E. cloacae* complex strains. The 2 ANC non-susceptible isolates were both carbapenemase-non-producing *E. cloacae* complex strains.

## Discussion

In this study, clinical isolates of CRE were sourced from 6 medical institutions in Japan to investigate the *in vitro* antibacterial activity of FNC and ANC and to compare them with other anti-CRE agents. Breakpoints were defined based on the CLSI and EUCAST guidelines. At a nacubactam concentration of 4 mg/L, both combinations inhibited the growth of at least 95% of CRE isolates across all strains tested (Table 2). Since September 2014, CRE infections have been comprehensively reported in Japan; a survey conducted from 2014 to 2024 indicated that *K. aerogenes*, *E. cloacae*, *K. pneumoniae* and *E. coli*^17^ are the predominant species, a trend also observed in the strains evaluated in this study.

In the evaluation of MBL-producing strains, 37.2% were susceptible to aztreonam alone (MIC ≤4 mg/L); however, aztreonam–nacubactam inhibited all strains at ≤2 mg/L, achieving 100% susceptibility based on the aztreonam breakpoint (≤4 mg/L). Although aztreonam is not hydrolysed by Class B enzymes (MBL), many Enterobacterales harbour AmpC enzymes on their chromosomes or plasmids,^18^ and many carbapenemase-producing strains express AmpC and/or Class A extended-spectrum β-lactamases.^19^ Aztreonam alone did not exhibit sufficient antibacterial activity against MBL-producing strains because of hydrolysis by these co-produced β-lactamases. When combined with nacubactam, which inhibits Class A and C β-lactamases, activity was restored. Similarly, when aztreonam was combined with avibactam, which also inhibits Class A and Class C enzymes commonly co-produced with MBLs, the susceptibility rate of MBL-producing strains increased to 100% (MIC ≤4 mg/L). The restoration of aztreonam activity against MBL-producing strains primarily reflects inhibition of co-produced Class A and C β-lactamases by nacubactam and avibactam.

Nacubactam also restored the activity of cefepime, increasing the susceptibility rate at a cefepime MIC of 2 mg/L from 3.2% to 86.5%. Notably, nacubactam possesses a unique dual mechanism of action that distinguishes it from other β-lactamase inhibitors such as avibactam and relebactam: in addition to inhibiting Class A and C β-lactamases, nacubactam demonstrates direct antibacterial activity through high-affinity binding to PBP2 in Gram-negative bacteria.^13–15^ Although nacubactam does not inhibit Class B β-lactamases, this intrinsic PBP2-binding activity may contribute to the restoration of cefepime activity against MBL-producing strains, complementing the inhibition of co-produced Class A and C enzymes. In contrast, ceftazidime–avibactam and imipenem–relebactam, which are not indicated for the treatment of MBL-producing organisms, did not demonstrate activity in this study. Neither avibactam nor relebactam inhibits MBLs, and unlike nacubactam, neither possesses intrinsic PBP-binding activity. β-lactam antibiotics exert their antibacterial activity by inhibiting PBPs, which are essential for bacterial cell-wall synthesis. However, differences in the occupancy patterns of PBPs lead to variations in the concentrations required for effective activity and morphological changes in bacteria.^20,21^ Carbapenems such as meropenem and doripenem bind simultaneously to PBP2 and PBP3 and exhibit strong antibacterial activities.^22,23^ For strains with reduced susceptibility to the co-administered β-lactam antibiotics, nacubactam may complement the overall antibacterial activity to levels that are comparable to carbapenems through its inhibition of PBP2. However, among CRE with the same MIC for cefepime, differences in resistance mechanisms have shown variations in *in vivo* efficacy.^24^ Detailed investigations, such as pharmacokinetic and pharmacodynamic analyses are therefore necessary to predict clinical efficacy.

Nacubactam enhances the antibacterial activity of cefepime and aztreonam against serine-type carbapenemase-producing bacteria. The combination of nacubactam with these β-lactam antibiotics inhibited the growth of all isolates of serine-type carbapenemase-producing strains at β-lactam concentrations of ≤2 or ≤4 mg/L. Similar results were observed when avibactam was combined with β-lactam antibiotics. In contrast, the susceptibility of these strains to imipenem–relebactam remained at 53.6%, with 11 of 28 strains (39.3%) showing MIC values ≥4 mg/L. Nacubactam and avibactam show broad-spectrum inhibitory activity against Class A enzymes (including KPC and GES), whereas relebactam's inhibitory activity is more specific to KPC-type carbapenemases.^2,3,25^ Importantly, relebactam has limited or no inhibitory activity against OXA-48-like carbapenemases.^2,3,26^ In this study, KPC was the most common serine-type carbapenemase (50.0%), followed by OXA-48, NMC/IMI, and other enzymes. The reduced activity of imipenem–relebactam against serine-type carbapenemase producers can be largely attributed to the presence of non-KPC carbapenemases, particularly OXA-48 producers. This finding is consistent with the known lack of relebactam activity against OXA-48-like enzymes.^2,3,7^ In contrast, nacubactam- and avibactam-based combinations demonstrated consistent activity across all serine-type carbapenemase classes, likely reflecting their broader inhibitory spectrum against Class A and C β-lactamases.

The isolate collection in this study reflects Japanese epidemiology, with MBL producers (predominantly IMP-type) accounting for 41.5% of CRE, whereas serine carbapenemases were uncommon (7.4%).^17^ This differs from other regions, where serine carbapenemases such as KPC and OXA-48-like enzymes are more prevalent.^26,27^ The high proportion of MBL producers influenced comparative agent performance: ceftazidime–avibactam and imipenem–relebactam showed limited activity, as these agents are not indicated for MBL-producing organisms. In contrast, nacubactam-based combinations demonstrated consistent activity across diverse resistance mechanisms through their dual action. Although results may differ in regions with higher prevalence of serine carbapenemases, this study suggests potential advantages of nacubactam combinations in MBL-endemic settings.

Among carbapenemase-non-producing CRE, MIC_50/90_ values for FNC and ANC were 0.25/4 mg/L and 0.5/4 mg/L, respectively. These values were comparable to those for ceftazidime–avibactam, aztreonam–avibactam and imipenem–relebactam. In *K. pneumoniae* and *E. cloacae* complex, carbapenem resistance in carbapenemase-non-producing strains has been attributed to the loss or mutation of porins, along with excessive production of ESBLs or AmpC.^28–30^ Porin deficiency particularly impairs the entry of imipenem among carbapenems into bacterial cells. In such strains, while β-lactamase inhibitors including avibactam, relebactam and nacubactam effectively inhibit ESBL and AmpC enzymes, the efficacy of imipenem–relebactam can be limited by reduced intracellular accumulation of imipenem due to porin deficiency or by efflux of relebactam.^7,31^ Information regarding resistance genes other than carbapenemase is unknown for the carbapenemase-non-producing carbapenem-resistant bacteria tested in this study; however, similar resistance mechanisms were inferred. Like avibactam and relebactam, nacubactam supports the restoration of the antibacterial activity of β-lactam antibiotics against carbapenemase-non-producing carbapenem-resistant strains by inhibiting the excessively produced ESBL and AmpC.

In this study, 2 carbapenemase-non-producing *E. cloacae* complex strains with elevated MIC values (FNC >8 mg/L; ANC >4 mg/L) were identified. Both strains also showed low susceptibility to the anti-CRE agents ceftazidime–avibactam and aztreonam–avibactam, but remained susceptible to imipenem–relebactam. Nacubactam, like mecillinam, acts through PBP2 binding to enhance the activity of β-lactams that preferentially target PBP3.^13–15,32^ Previous laboratory studies have shown that experimental strains with reduced nacubactam susceptibility, similar to mecillinam-resistant laboratory strains, can develop compensatory mechanisms for PBP2 inhibition.^33^ Whether similar mechanisms account for the elevated MIC values in clinical isolates remains to be determined. Furthermore, all strains with elevated FNC and ANC MIC values (FNC >8 mg/L; ANC >4 mg/L) in this study showed low susceptibility to cefepime, aztreonam, meropenem, ceftazidime–avibactam and aztreonam–avibactam while maintaining susceptibility to imipenem,^22,34^ which strongly binds to PBP1A and PBP1B, and imipenem–relebactam. This pattern indicates that evaluating PBP-binding profiles may help elucidate the mechanisms underlying reduced susceptibility to nacubactam combinations.

Despite these important findings, this study has some limitations. First, the *in vitro* design of the study, without PK/PD analyses or clinical correlations, constrains the translation of MICs to patient efficacy. Second, testing was conducted across 6 laboratories using different carbapenemase-typing methods; therefore, some inter-laboratory variability and misclassification may remain. Third, CLSI/EUCAST breakpoints for cefepime–nacubactam and aztreonam–nacubactam are not yet defined; therefore, we have only presented MIC distributions for these combinations, limiting direct clinical interpretability. Fourth, we did not systematically assess other resistance determinants (such as porins, PBP3 insertions, ESBL/AmpC and efflux), precluding mechanistic attribution for resistance to these combinations in a subset of isolates. Fifth, the isolate collection was enriched for IMP-type producers, which may limit generalizability. Sixth, cefiderocol was not included in this comparative analysis because its susceptibility testing requires specialized iron-depleted media (ID-CAMHB), precluding direct comparison with other agents under identical conditions. Overall, these data demonstrate that FNC and ANC exhibit excellent *in vitro* activity against CRE, independent of carbapenemase presence. Nacubactam is effective against local IMP-type carbapenemase producers, as well as globally significant NDM-type, OXA-48-type and KPC-type strains, making nacubactam combinations a promising therapeutic option for CRE infections in Japan and worldwide. Further exploration is warranted to correlate the *in vitro* antibacterial activity and clinical efficacy, the mechanisms of action of enhancers not related to β-lactamase inhibition, and the resistance mechanisms of clinically resistant strains. Understanding these factors is crucial for optimizing the use of nacubactam combinations in clinical settings.

## Transparency declarations

Katsunori Yanagihara and Hiroshige Mikamo received research funding from Meiji Seika Pharma for separate studies involving nacubactam. Katsunori Yanagihara, Kazuhiro Tateda, Satoshi Takahashi, Hiroki Ohge and Hiroshige Mikamo received honoraria or fees from Meiji Seika Pharma for lectures, manuscript preparation unrelated to nacubactam, advisory board participation related to nacubactam, or medical expert services related to nacubactam or other matters. Yohei DOI received fees for medical expert services from Meiji Seika Pharma. Koju Itahashi and Hayato Okade are employees of Meiji Seika Pharma.
